# The Role of Polysomnography for Children with Attention-Deficit/Hyperactivity Disorder

**DOI:** 10.3390/life15040678

**Published:** 2025-04-21

**Authors:** Chien-Heng Lin, Po-Yen Wu, Syuan-Yu Hong, Yu-Tzu Chang, Sheng-Shing Lin, I-Ching Chou

**Affiliations:** 1 Division of Pediatric Pulmonology and Critical Care Medicine, China Medical University Children’s Hospital, Taichung 404327, Taiwan; lch227@ms39.hinet.net; 2 Department of Biomedical Imaging and Radiological Science, College of Health Care, China Medical University, Taichung 404327, Taiwan; 3 Division of Pediatric Neurology, China Medical University Children’s Hospital, Taichung 404327, Taiwan; 036088@tool.caaumed.org.tw (P.-Y.W.); dazingdog@hotmail.com (S.-Y.H.); 004298@tool.caaumed.org.tw (Y.-T.C.);

**Keywords:** attention-deficit/hyperactivity disorder, polysomnography, sleep problem, children, sleep-disordered breathing

## Abstract

**Objective:** Attention-Deficit/Hyperactivity Disorder (ADHD) is a common neurodevelopmental disorder in children, characterized by inattention, hyperactivity, and impulsive behavior. In recent years, studies have shown that patients with ADHD often experience sleep problems, raising clinical interest in the potential role of polysomnography (PSG) in the diagnosis and management of ADHD. This study examines polysomnographic findings in children with ADHD who present with diverse sleep complaints. **Methods:** A cohort of children aged younger than 18 years, diagnosed with ADHD based on DSM-5 criteria, underwent overnight polysomnography. The study assessed various sleep parameters, including sleep latency, sleep efficiency, total sleep time, and the presence of sleep-disordered breathing. **Results:** A retrospective analysis was conducted on 36 children (29 boys and 7 girls) aged 6 to 14 years, diagnosed with ADHD, who underwent polysomnography between 2021 and 2024. Polysomnographic findings revealed that 77.78% of the children demonstrated significant snoring. Furthermore, 50.0% were diagnosed with obstructive sleep apnea syndrome (OSAS). In addition, eight children exhibited parasomnias. Among them, six had bruxism, three were diagnosed with periodic limb movement disorder (PLMD), and two experienced sleep talking. Other notable sleep-related conditions included two cases of narcolepsy, one case of prolonged sleep onset latency, and one case of central apnea syndrome. Total sleep time (TST) was significantly longer in females compared to males (400.71 ± 32.68 min vs. 361.24 ± 41.20 min, *p* = 0.0215), whereas rapid eye movement (REM) latency was longer in males compared to females (118.62 ± 55.60 min vs. 78.57 ± 27.82 min, *p* = 0.0194). These findings highlight the high prevalence of sleep-disordered breathing (SDB) in children with ADHD who present with sleep disturbances. Furthermore, sleep quality, as indicated by longer TST and shorter REM latency, appears to be better in females with ADHD. **Conclusions:** The findings of this study underscore the critical role of polysomnography (PSG) in the assessment of children with ADHD. PSG provides an objective evaluation of sleep abnormalities commonly associated with ADHD, which may influence symptom manifestation and treatment outcomes. Notably, the results suggest that females with ADHD exhibit better sleep quality, as indicated by longer total sleep time (TST) and shorter rapid eye movement (REM) latency compared to males. We recommend incorporating polysomnography (PSG) into the comprehensive assessment of children with ADHD who present with significant sleep disturbances. Further research is warranted to investigate the impact of targeted interventions for sleep abnormalities on ADHD symptoms, prognosis, and potential sex-specific differences.

## 1. Introduction

Attention-Deficit/Hyperactivity Disorder (ADHD) is one of the most common neurodevelopmental disorders of childhood, with an estimated global prevalence of approximately 5% to 7% among school-aged children [1,2]. It is characterized by persistent and developmentally inappropriate levels of inattention, hyperactivity, and impulsivity, which significantly impair academic performance, interpersonal relationships, sleep quality, and overall quality of life. In addition, the symptoms of ADHD and sleep disorders overlap; therefore, in recent years, an increasing body of research has highlighted the link between ADHD and sleep disturbances [3,4]. Emerging evidence highlights that sleep disturbances are not only prevalent but also clinically significant in children with ADHD. Studies report that up to 70% of children with ADHD, as compared to 20–30% of typically developing children, experience sleep disturbances [5]. Children with ADHD experience difficulties such as sleep-disordered breathing (SDB), periodic limb movement sleep disorder (PLMSD), insomnia, increased night-time awakenings, and shorter total sleep duration [6]. Up to 56.8% of children with ADHD experience SDB, compared to approximately 19.3% in the general pediatric population [7]. PLMSD is reported in 26% of children with ADHD versus a rate of about 1.2–5% in typically developing children [8,9]. Insomnia symptoms are present in up to 70% of children with ADHD, compared to a rate of 19.3% in children without ADHD [10].

Children with ADHD frequently experience sleep disturbances, which can exacerbate ADHD symptoms and complicate treatment strategies [3,4,5,6,7,8,9,10]. However, the exact link or pathogenesis between sleep problems and ADHD is still not clear.

Polysomnography (PSG) is the gold standard for evaluating sleep architecture and diagnosing sleep-related disorders. It provides comprehensive and objective assessments of various physiological parameters, including sleep stages, respiratory events, limb movements, and arousals [4,5,6,7,8,9]. While PSG is routinely used to assess conditions such as obstructive sleep apnea syndrome (OSAS) and PLMSD, its role in the clinical evaluation of children with ADHD has been relatively under-investigated [11,12,13,14,15,16]. Several studies have suggested that PSG may reveal subtle abnormalities in sleep architecture among children with ADHD, such as reduced REM sleep, increased sleep latency, or elevated arousal indices, which are not always apparent through clinical history or subjective sleep questionnaires. However, the underlying mechanisms linking ADHD and sleep pathology remain poorly understood, with hypotheses ranging from dopaminergic dysfunction and circadian rhythm disturbances to shared genetic and neurobiological pathways. Given the growing recognition of the interplay between ADHD and sleep, there is a need for more systematic exploration of PSG findings in affected children. Furthermore, sex-based differences in ADHD presentation—including behavioral symptoms, comorbidities, and neurobiological correlates—suggest that sleep profiles may also differ by gender, yet this area remains insufficiently studied. The primary hypothesis of this study is that children with ADHD who present with sleep-related complaints will exhibit measurable abnormalities in PSG parameters and that these abnormalities may differ between boys and girls. The research objectives are as follows: (1) To characterize PSG findings in children diagnosed with ADHD and sleep complaints; (2) to compare PSG parameters between male and female participants to explore potential sex-based differences in sleep architecture and disturbances; and (3) to assess the potential utility of PSG in informing clinical management strategies for children with ADHD, particularly in identifying coexisting sleep disorders that may exacerbate ADHD symptoms.

## 2. Methods

### 2.1. Participants

This retrospective study analyzed a cohort of pediatric patients under the age of 18 years who were diagnosed with ADHD according to the criteria outlined in the Diagnostic and Statistical Manual of Mental Disorders, Fifth Edition (DSM-5). All participants were referred to a sleep laboratory due to reported sleep disturbances and underwent overnight PSG between 2021 and 2024. All eligible cases within this timeframe were included to ensure a comprehensive analysis of sleep patterns in this population. Epworth sleepiness scale (ESS) scores were recorded. The exclusion criteria included intellectual disability, history of trauma, congenital abnormalities, and comorbid psychiatric disorders. The study protocol was approved by the Institutional Review Board of China Medical University Hospital (CMUH109-REC1-067).

### 2.2. Polysomnography Procedure

Polysomnographic assessments were conducted at an accredited sleep center under standardized conditions. Electroencephalography (EEG), electrooculography (EOG), electromyography (EMG), electrocardiography (ECG), nasal airflow monitoring, pulse oximetry, thoracoabdominal respiratory effort assessment, and audio/video recordings were included in the PSG protocol. Sleep architecture and quality were evaluated by measuring sleep latency, sleep efficiency, total sleep time (TST), rapid eye movement (REM) sleep percentage, and sleep stage. Additionally, respiratory parameters, including arousal, apnea–hypopnea index (AHI), and sleep positioning, were recorded and analyzed.

Limb movement abnormalities, including PLMD, were assessed using EMG recordings of the lower extremities. The presence of parasomnias such as bruxism and sleep talking were documented based on video findings of PSG and parental reports. PSG parameters such as AHI, TST, sleep efficiency, sleep onset latency, and REM sleep duration were specifically compared between male and female participants to assess potential gender-related differences.

### 2.3. Statistical Analysis

Data were analyzed using PASW Statistics version 18.0 (SPSS Inc., Chicago, IL, USA), with continuous variables expressed as the mean ± standard deviation (SD). To compare polysomnographic (PSG) parameters between male and female participants, we utilized the Mann–Whitney U test, a nonparametric statistical method appropriate for assessing differences between two independent groups when data are not normally distributed. This test was chosen due to its robustness in handling skewed distributions and ordinal data, ensuring a reliable comparison of PSG metrics across sexes. We also used Fisher’s Exact Test for categorical data analysis for sleep disorders and comorbidities between males and females due to the small sample size in each category. A *p*-value less than 0.05 indicates a statistically significant difference.

## 3. Results

A total of 36 children (29 boys, 7 girls) aged between 6 and 14 years were included in the study. ESS scores demonstrated no significant differences between male and female participants (5.52 ± 5.49 vs. 4.86 ± 3.91, *p* = 0.7205).

### 3.1. Polysomnographic Findings and Sleep Architecture (Table 1)

The analysis of sleep parameters revealed significant gender-based differences. Total sleep time (TST) was significantly longer in females compared to males (400.71 ± 32.68 vs. 361.24 ± 41.20, *p* = 0.0215), and sleep latency was significantly shorter in girls than in boys (118.62 ± 55.60 vs. 78.57 ± 27.82, *p* = 0.0194). Other parameters of PSG did not reach statistical significance.

**Table 1:** Polysomnographic findings and sleep architecture between boy and girl.

Parameter	Boy (*n* = 29; Mean + SD)	Girl (*n* = 7; Mean + SD)	*p*-Value	Effect Size
Age	9.21 ± 2.47	8.14 ± 3.34	0.3963	0.40
AHI	4.98 ± 12.09	1.76 ± 1.68	0.5222	0.2928
BMI	18.65 ± 4.47	17.88 ± 5.25	0.5894	0.1667
REM	4.48 ± 4.76	4.14 ± 0.69	0.1990	0.0785
SPT	396.79 ± 80.02	431.57 ± 36.85	0.0501	−0.4584
TST	361.24 ± 41.20	400.71 ± 32.68	0.0215 *	−0.9910
Sleep Efficiency	0.8951 ± 0.0676	0.9210 ± 0.0486	0.3186	−0.4006
Sleep Latency	16.21± 14.11	16.80 ± 14.64	0.9220	−0.0415
REM Latency	118.62 ± 55.60	78.57 ± 27.82	0.0194 *	0.7733
N1	8.89 ± 6.29	6.51 ± 4.64	0.3791	0.3945
N2	46.89 ± 9.57	48.04 ± 12.48	0.8730	−0.1134
N3	17.15 ± 9,13	18.82 ± 9.82	0.8416	−0.1804
REM Percentage	19.86 ± 6.149	47.13 ± 70.15	0.1990	−0.9092
Arousals	107.33 ± 65.14	119.14 ± 69.26	0.9045	−0.1792
Supine	46.76 ± 23.64	38.33 ± 16.44	0.5488	0.3740
Right	25.45 ± 15.59	33.44 ± 20.02	0.2007	−0.4855
Left	19.91 ± 16.30	22.94 ± 16.22	0.6307	−0.1861
Prone	7.89 ± 12.43	5.13 ± 8.72	0.6384	0.2327
Limb Movements	1.54 ± 1.68	2.30 ± 2.43	0.5230	−0.4142

AHI = apnea hypopnea index; BMI = body mass index; REM = rapid eye movement; SPT = sleep period time; TST = total sleep time. * = *p* < 0.05

Regarding sleep positioning, there was no significant gender difference in the proportion of time spent in supine, right, left, or prone positions during sleep. The number of limb movements was slightly higher in girls than in boys (2.30 ± 2.43 vs. 1.54 ± 1.68), but this difference was not statistically significant (*p* = 0.5230).

### 3.2. Prevalence of Sleep Disorders (Table 2)

A high prevalence of snoring was observed in the study cohort, affecting 77.78% (28/36) of children, with no significant gender difference (*p* = 1). OSAS was identified in 50.0% (18/36) of the participants, and all cases were the mild type, except for one case, which was the moderate type (AHI = 5.6), again without a significant gender difference (*p* = 1).

**Table 2:** Comparison of prevalence of sleep disorders and comorbidities between male and female.

Sleep Disorder	Male (*n* = 29)	Female (*n* = 7)	Total (*n* = 36)	*p*-Value	Odds Ratios
Snoring	22	6	28 (77.78%)	1	0.5238
OSAS	14	4	18 (50%)	1	0.7000
Parasomnias	4	4	8 (22.22%)	0.0301 *	0.1200
Bruxism	3	3	6 (16.67%)	0.0732	0.1538
Sleep talking	1	1	2 (5.56%)	0.3555	0.2143
PLMSD	1	2	3 (8.34%)	0.0902	0.0893
Prolonged sleep onset	0	1	1 (2.78%)	0.1944	infinite
Narcolepsy	2	0	2 (5.56%)	1	infinite
Central apnea	1	0	1 (2.78%)	1	infinite
Comorbidity					
Allergic history	7	1	8 (22.22%)	1	1.9090
Asthma	4	0	4 (11.11%)	0.5658	infinite
Tourette’s disorder	1	0	1 (2.78%)	1	infinite
Asperger syndrome	1	0	1 (2.78%)	1	infinite

OSAS = obstructive sleep apnea syndrome; PLMSD = periodic limb movement sleep disorder. * = *p* < 0.05.

In our analysis comparing the prevalence of sleep disorders and comorbidities between males (*n* = 29) and females (*n* = 7), significant differences were observed in the occurrence of parasomnias (*p* = 0.0301), with a higher prevalence in girls. While other sleep disorders, such as snoring (77.78%), OSAS (50%), bruxism (16.67%), and PLMSD (8.34%), were reported in both groups, no statistically significant sex-based differences were detected (all *p* > 0.05).

### 3.3. Comorbidities (Table 2)

Among the study cohort, 22.22% (8/36) of participants had a history of allergic conditions, with no significant gender differences (*p* = 1). Asthma was reported in 11.11% (4/36) of cases, exclusively in males (*p* = 0.5658). Other comorbidities included Tourette’s disorder (2.78%) and Asperger’s syndrome (2.78%), each diagnosed in a single male participant (*p* = 1).

## 4. Discussion

The findings of this study highlight the high prevalence of sleep abnormalities in children with ADHD, especially snoring (79.4%) and OSAS (50%), reinforcing the importance of sleep evaluation in this population. The significant occurrence of SDB (snoring and OSAS) among children with ADHD suggest that sleep dysfunction may contribute to the persistence and severity of ADHD symptoms [12,13,14,15,16].

However, Rosalia Silvestri et al. reported that in 55 children with ADHD (47 M, 8 F; mean age = 8.9 y), most had disturbed, fragmentary sleep at night, and their complaints included motor restlessness (50%), sleep walking (47.6%), night terrors (38%), confusional arousals (28.5%), snoring (21.4%), and restless legs syndrome (RLS) (11.9%) [15]. The higher snoring rate in our study (79.4%) compared to Silvestri et al.’s 2009 study (21.4%) may be due to several factors. First, parents might more readily observe and report snoring, a prominent and audible symptom, over other sleep disturbances, such as motor restlessness or parasomnias, leading to higher reported rates in our cohort. Second, at our institution, pulmonologists may be more inclined to recommend PSG for children with ADHD who present with snoring, whereas other sleep disturbances might not prompt the same level of investigation. This referral bias could contribute to the increased detection of snoring in our study population.

OSAS is a condition characterized by repetitive episodes of upper airway obstruction during sleep, leading to fragmented sleep, hypoxia, and excessive daytime sleepiness. The relationship between ADHD and OSAS is complex as both conditions share overlapping symptoms, such as inattention, hyperactivity, and impulsivity. These results align with previous research suggesting a bidirectional relationship between ADHD and sleep disturbances [5,6,7,8,9,10,11,12,13,14].

Children with OSAS often exhibit cognitive and behavioral deficits, which may exacerbate ADHD symptoms. The disruption of sleep architecture caused by OSAS can lead to poor sleep quality, which in turn affects daytime functioning. Furthermore, untreated OSAS has been linked to impaired academic performance, emotional dysregulation, and an increased risk of comorbid psychiatric disorders in children with ADHD.

Apart from OSAS, this study also identified other sleep disturbances, including PLMSD, parasomnias (such as bruxism and sleep talking), and prolonged sleep onset latency in some cases. PLMSD, which involves repetitive limb movements during sleep, can cause frequent nocturnal arousals, leading to daytime fatigue and exacerbating ADHD symptoms. Similarly, parasomnias, including bruxism, may contribute to sleep fragmentation and reduced sleep efficiency in affected children.

The high prevalence of sleep disturbances in children with ADHD underscores the need for systematic sleep evaluations, including PSG, in this population. Proper diagnosis and management of sleep disorders, particularly OSAS, may significantly reduce ADHD symptoms, prevent unnecessary long-term methylphenidate (MPH) usage and potential drug side effects, and improve overall quality of life [16]. In our study, there was only one patient who had moderate OSAS, and none of them received the surgical intervention for OSAS.

Children with ADHD exhibit distinct sleep architecture changes, including prolonged REM sleep duration and an increased number of sleep cycles. These findings suggest underlying neurobiological mechanisms involving altered monoaminergic activity and cortical inhibitory dysfunction [17].

In another study by Chin WC et al., children with ADHD experienced a wide range of sleep disturbances, including a higher AHI and higher hypopnea counts, reduced slow-wave sleep, increased sleep fragmentation, and various parasomnias. In this study, the PSG data showed significantly increased TST (*p* = 0.005) and decreased periodic limb movement index (PLMI) (*p* = 0.031) after 6-month MPH treatment [18]. Some studies in the literature have reported that parasomnias are more frequently observed in children with ADHD, but these studies do not provide detailed analyses based on gender [16,19].

A key finding in this study was the significant gender differences in sleep parameters observed in children with ADHD. Girls demonstrated a significantly longer TST and a shorter REM latency compared to boys, and the prevalence of parasomnias is higher in females. These differences suggest potential variations in sleep architecture between males and females with ADHD, which could have implications for symptom expression and treatment strategies.

The increased TST in girls may indicate that female children with ADHD require or achieve longer sleep duration, potentially due to differences in the underlying neurophysiology of ADHD. Longer sleep duration may help mitigate some of the behavioral symptoms of ADHD in girls, whereas boys, who had shorter total sleep duration, may experience greater daytime impairments due to insufficient restorative sleep.

Additionally, the shorter REM latency in girls is particularly notable. REM sleep is crucial for cognitive function, emotional regulation, and memory consolidation. A longer REM latency in boys may contribute to greater difficulties with emotional control, executive function, and attentional regulation, which are core features of ADHD. Given that REM sleep plays a critical role in neurodevelopment, differences in REM sleep duration between genders might influence how ADHD symptoms manifest differently in boys and girls.

The observed gender differences could also be influenced by hormonal, genetic, and environmental factors. For example, previous research has suggested that estrogen and other sex hormones may play a role in sleep regulation and may contribute to longer sleep duration and greater REM sleep percentage in females [20]. Moreover, progesterone is known to enhance sleep duration and quality by promoting slow-wave sleep (SWS). Moreover, boys with ADHD tend to have a higher prevalence of externalizing behaviors, which might result in higher physiological arousal, leading to shorter sleep durations and reduced REM sleep.

In our study, only one female adolescent participant (older than 12 years of age) was included, limiting the potential impact of hormonal influences on sleep architecture. Further research with larger sample sizes is necessary to determine the extent to which gender differences influence PSG parameters or whether these gender differences in sleep parameters translate to differences in ADHD symptomatology and treatment responses. Understanding the interplay between sleep architecture and ADHD symptoms in boys and girls could guide more tailored treatment approaches, such as behavioral interventions and pharmacological adjustments, to optimize sleep quality and improve overall ADHD management.

Children with ADHD and sleep disturbances may exhibit increased daytime inattention, impulsivity, and emotional dysregulation due to fragmented sleep and reduced sleep quality. The presence of PLMSD, bruxism, and parasomnias further underscores the need for sleep assessments in ADHD management. Additionally, the gender differences observed in TST and REM latency suggest potential variations in sleep physiology between boys and girls with ADHD, which may have implications for treatment and prognosis.

## 5. Limitations

Despite the valuable findings of this study, several limitations should be acknowledged. First, the sample size of 36 children is relatively small, which may limit the generalizability of the results. It is important to note that our study included only seven female participants. This small sample size limits the statistical power and generalizability of our findings related to female subjects. Therefore, these results should be interpreted with caution and considered preliminary. Further prospective studies with larger cohorts are necessary to validate and expand upon these observations. Second, in this study, we employed a retrospective design, which inherently limits the ability to establish causal relationships between sleep disturbances and ADHD symptomatology. Future prospective studies with follow-ups are needed to determine whether addressing sleep disturbances leads to significant improvements in ADHD symptoms. Third, although PSG provides objective data on sleep patterns, it is a single-night evaluation, which may not capture the full spectrum of sleep variability in children with ADHD. Home-based or multi-night PSG assessments may provide more comprehensive insights into sleep disturbances. Fourth, the study lacks control groups consisting of typically developing children or children with other neurodevelopmental disorders. A comparison group would help determine whether the observed sleep abnormalities are unique to ADHD or are common in other pediatric populations.

Finally, the potential influence of medication use on sleep parameters was not controlled in this study. Many children with ADHD take stimulant medications, which can affect sleep patterns, potentially confounding the observed PSG findings. This lack of information may have influenced the PSG parameters and should be considered when interpreting the results. Future research should explore the clinical implications of these sleep alterations and potential therapeutic strategies to optimize sleep quality in ADHD populations.

## 6. Conclusions

Given the high prevalence of sleep-disordered breathing (SDB) in ADHD, affecting nearly 80% of patients, PSG should be considered as part of a comprehensive ADHD evaluation, particularly in patients reporting significant sleep disturbances. Notably, TST tends to be significantly longer but REM latency tends to be significantly shorter in girls than in boys, highlighting potential gender differences in sleep architecture. Including PSG in ADHD evaluations can help optimize treatment strategies and improve overall patient outcomes. These differences may have clinical implications when selecting or adjusting treatment approaches. For example, girls may require more cautious titration of pharmacological interventions, and behavioral strategies targeting sleep hygiene may be tailored based on individual sleep profiles. Furthermore, PSG assessments before and after treatment can provide valuable insights into therapeutic effectiveness.

## Data Availability

Datasets are available on request.
